# Prevalence, infection intensity and associated factors of soil transmitted helminths among primary school children in Gurage zone, South Central Ethiopia: a cross-sectional study design

**DOI:** 10.1186/s13104-019-4254-8

**Published:** 2019-04-16

**Authors:** Habtamu Weldesenbet, Abdulsemed Worku, Teha Shumbej

**Affiliations:** 1 Department of Medical Laboratory Science, College of Medicine and Health Sciences, Wolkite University, P.O. Box 07, Wolkite, Ethiopia; 2 Department of Medicine, College of Medicine and Health Science, Wolkite University, Wolkite, Ethiopia

**Keywords:** Soil transmitted helminthes, Intestinal parasites, School children, Gurage zone, Prevalence, Ethiopia, Wolkite

## Abstract

**Objective:**

The aim of this study was to determine the prevalence of soil transmitted helminthes among primary school children. School based cross-sectional study design was employed. A total of six hundred study subjects were selected by a multistage sampling method. Fresh stool specimens were collected using clean, dry and wide mouthed labeled stool cups. It was processed by Kato-Katz technique. The data were analyzed using SPSS version 20 and p-value < 0.05 was considered statistically significant.

**Result:**

The overall prevalence of soil transmitted helminthes was 57 (9.5%). *Hookworm* was the most prevalent helminthes species isolated (4.2%) followed by *A. lumbricoide* (3%). The prevalence of *Taenia* species, *T. trichiura*, *H. nana* and *E. vermicularis* were; 1.2%, 0.5%, 0.7% and 0.8% respectively. The prevalence of the Soil transmitted helminthes infection was low and all cases of Soil transmitted infections in this study were with low infection intensity. This might be due to the preventive chemotherapy given to the school children.

**Electronic supplementary material:**

The online version of this article (10.1186/s13104-019-4254-8) contains supplementary material, which is available to authorized users.

## Introduction

Intestinal helminthes infections are among the most common infections in school children. Globally, an estimated number of 878 million school aged children and 386 million Preschool aged children are at risk [1]. The prevalence of soil transmitted helminthes (STH) infection is mainly attributed to their chronic and insidious impact on the health quality of life those infected rather than the mortality they cause. Heavy infection of STH impairs physical growth and brain development, and are the causes of micronutrient deficiency including iron deficiency anemia leading to poor school performance and reduced work productivity in adults and adverse pregnancy outcomes [2]. The soil transmitted helminthes strategic plan 2011–2020 has outlined four miles stones for global control of soil-transmitted helminthes infection [3]. School children aged 5–15 years suffer the highest infection rate and worm burden due to poor personal hygiene and low sanitation [4, 5].

Soil-transmitted helminthes are highly abundant in Ethiopia; *hookworm* infects 11 million people, thus Ethiopia bears 5.6% which was the third highest burden in Sub-Saharan Africa and *T. trichiura*, with 21 million people, which was the 4th highest (13%) of the diseases burden in Sub-Saharan Africa [6, 7]. Similarly *A. lumbricoide* estimated to infect 26 million people which were the second highest burden (15%) of the overall burden in Sub-Saharan Africa [8, 9].

Study conducted in Babile Town, Ethiopia also showed Prevalence of hookworm infection rate was significantly lower in children who wore shoes [10]. Study conducted in Bahir Dar, Ethiopia also showed having dirty hands’ fingernail and untrimmed hands’ fingernail were positively associated with the overall prevalence of intestinal helminthes infection [11].

In the 2013–2014 Ethiopian national mapping of infections of soil transmitted helminths and association with School Water, Sanitation, and Hygiene was assessed alongside infection intensity in school children, and the result have shown that *A. lumbricoides*, *T. trichiura*, and *hookworm* were 13.3%, 7.8%, and 7.4% respectively. At least one case of each of those helminths was found in 75.4%, 51.7%, and 52.0% of these schools, respectively [12].

Preventive Chemotherapy treatment was began in the study area since 2015. Three rounds of preventive chemotherapy treatments were given in the study area till 2017. Even though there was no study done in the study area before the preventive chemotherapy treatment began, the national mapping done 2013/2014 has shown that prevalence of soil transmitted helminths in the country was high. Therefore it is important to determine the prevalence and infection intensity of soil transmitted helminths to see preventive chemotherapy treatment outcomes. Therefore, this study was intended to investigate the prevalence, infection intensity and associated factors of Soil transmitted helminths among primary school children in Gurage zone, South Central Ethiopia after three rounds of preventive chemotherapy treatment.

## Main text

### Methods and materials

#### Study design and area

A cross sectional study design was performed among primary school children in Gurage zone to estimate the prevalence of soil transmitted helminthes and associated factors. There was lack of water supply in the study area and many people in the community, including school children were exposed for soil transmitted helminthes due to poor personal hygiene.

#### Sample size and sampling technique

Three districts were selected using purposive method based on previous data of soil transmitted helminthes. From the three districts six schools were selected based on population density, ecological zone and risk factors. Proportionate sample was allocated to each school based on school population density. In each school the sample was distributed according to the number of the students in each grade and section. The registration list was used as the sampling frame. The required sample size for this study was estimated using the general formula for single population proportion [13] and considering 36.7% [14] prevalence rate in the population, 95% confidence level and 5% margin of error with 1.5 design effect. Accordingly the following formula was applied:

$${\text{n}} = \frac{{{\text{Z}}^{ 2} {\text{P}}\left( { 1- {\text{P}}} \right)}}{{{\text{d}}^{ 2} }} = {{\left( { 1. 9 6} \right)^{ 2} 0. 3 7\left( { 1- 0. 3 7} \right)} \mathord{{\vphantom {{\left( { 1. 9 6} \right)^{ 2} 0. 3 7\left( { 1- 0. 3 7} \right)} {\left( {0.0 5} \right)^{ 2} }}}}/ {\left( {0.0 5} \right)^{ 2} }} = 3 5 8* 1. 5= 5 3 7.$$

Assumptions: P = prevalence rate, d = margin of sampling error tolerated between the sample and population 5%, α = critical value at 95% confidence interval of certainty (1.96), n = sample size, Z = 95% confidence level.

Considering 10% for anticipated non-response rate and design effect of 1.5, the sample size is 600.

#### Inclusion criteria

The participants whose guardian or parent signed a written informed consent and all children who were available and registered in their school during the study period and who gave stool sample were included in the study.

#### Exclusion criteria

Participants who were on anti-helminthic drug or treatment within 2 weeks prior to data collection and study subjects who were unable to provide a stool specimen at the time of sampling were excluded from the study.

### Data collection and processing

#### Socio-demographic data and risk factor assessment of study participants

The questionnaire was prepared in English and translated to local language Guragegna and checked for fitness. Pre-test was done in 5% of school children that were not included in the study. Appropriate Correction was made according to the result. Parents or guardian of the school children participated in the study were interviewed with the pre-tested questionnaire to collect the demographic data and associated risk factors.

#### Parasitological techniques

Clean, dry and tightly screwed stool cup was given to each study subjects. Sufficient amount of stool sample was collected from each student. The stool samples were processed by Medical Laboratory Technologists using the standard Kato-Katz technique [15].

In the Kato-Katz technique feces were pressed through a mesh screen to remove large particles. A portion of the sieved sample was then transferred to the hole of a template on a slide. After filling the hole, the template was removed and the remaining sample (approx. 50 mg) was covered with a piece of cellophane soaked in glycerol (glycerine). The glycerol ‘clears’ the faecal material from the eggs.

A microscopic examination was done to identify the parasites and infection intensity within 30 min of collection. The slide smears were examined using 10× and 40× objectives. The eggs were then counted and the number was calculated per gram (g) of feces to determine the intensity of the infection in each slide according to the WHO bulletin in 1993 [16].

#### Data quality control

Training was given to data collectors and Medical Laboratory technologists before the start of data collection. The questionnaire was checked for completeness and any incomplete questionnaire was corrected under supervision during data collection. Two slides were prepared from each sample. The slides were examined by two Medical Laboratory Technologists independently first with Low (10×) objective followed by the middle (40×) objective. Ten percent of the samples were randomly selected and re-checked by Senior Medical Laboratory Technologists.

#### Data analysis and Interpretation

The data were entered into SPSS version 20.0 for statistical analysis. The demographic data were described by descriptive statistics. The prevalence of soil transmitted helminthes was determined. Bivariate analysis was done and 95% confidence interval was calculated. The p-value of < 0.25 in bivariate was taken to build adjusted binary logistic regression analysis. Logistic regression model with backward elimination was taken. Variables that were associated with soil transmitted helminthes at < 0.05 were retained in the model.

### Result

A total of 600 school children were included in this study. From these study subjects, 349 (58.17%) were males and 251 (41.83%) were females. The mean age of the study subjects was 10.48 years with a minimum of 4 years and maximum of 17 years (Table 1).

**Table 1:** Socio-demographic characteristics of study participants in Gurage zone southern Ethiopia (January to December 30, 2017) (n = 600)

	Frequency	Percent (%)
Gender of the child		
Male	349	58.17
Female	251	41.83
Total	600	100.0
Age		
Mean of age	10.48	
Minimum age	4	
Maximum age	17	
Age group of children		
< 6	9	1.5
6–10	299	49.8
11–15	264	44.0
> 15	28	4.7
Total	600	100.0
Residence of the child		
Urban	317	52.83
Rural	283	47.27
Total	600	100.0
Mother’s highest level of education		
Illiterate	277	46.17
Informal education	1	0.17
Primary 1_8	281	46.83
Secondary 9_12	31	5.17
Diploma	10	1.66
Total	600	100.0
Districts of the child		
Abeshge	297	49.50
Cheha	103	17.17
Kebena	200	33.33
Total	600	100.0
Ethnicity of the child		
Gurage	265	44.17
Amhara	260	43.33
Oromo	54	9.0
Tigre	3	0.5
Other	18	3.0
Total	600	100.0

The overall prevalence of soil transmitted helminthes among school children was 57 (9.5%), among them 35 (10.03%) were males and 23 (9.16%) were females. Higher prevalence was observed among males than females (10.03% vs. 9.16%). With regard to specific age group, school children in the age group of < 6 years were more infected than 6–10 years and 11–15 years of age groups of the study subjects, 22.22%, 10.37% and 9.09% respectively. None of the study subjects with age group of > 15 years have soil transmitted helminthes infection (Table 2). *Hookworm* was the highest soil transmitted helminthes infection (4.2%) followed by *A. lumbricoide* (3%) while *Taenia* species, *E. vermicularis*, *H. nana* and *T. trichiura* were 1.2%, 0.8%, 0.7% and 0.5% respectively (Additional file 1: Table S1).

**Table 2:** The overall prevalence of the soil transmitted helminthes and the significance of associated risk factors among school children in Gurage zone southern Ethiopia (January to December 30, 2017) (n = 600)

Variables	Soil transmitted helminthes			p-value
	No	Yes	Total	
Gender				
Male	314 (89.97%)	35 (10.03%)	349 (58.17)	> 0.05
Female	228 (90.84%)	23 (9.16%)	251 (41.83%)	1
Age group				
< 6	7 (77.78%)	2 (22.22%)	9 (1.5%)	1
6–10	268 (89.63%)	31 (10.37%)	299 (49.83%)	> 0.05
11–15	240 (90.91%)	24 (9.09%)	264 (44.0%)	> 0.05
> 15	28 (100%)	0 (0%)	28 (4.67%)	> 0.05
Districts				
Abeshege	271 (91.25%)	26 (8.75%)	297 (49.50%)	> 0.05
Kebena	182 (91.24%)	18 (8.76%)	200 (33.33%)	> 0.05
Cheha	88 (86.27%)	14 (13.73%)	103 (17.00%)	1
Mother’s education				
Illiterate	254 (91.67%)	23 (8.30%)	277 (46.17%)	> 0.016
Informal	1 (100%)	0 (0%)	1 (0.17%)	> 0.05
Primary	249 (89.89%)	31 (11.07%)	280 (46.67%)	> 0.05
Secondary	33 (100%)	0 (0%)	33 (5.5%)	> 0.05
University	6 (66.67%)	3 (33.33%)	9 (1.5%)	1
Play in the soil				
Yes	496 (90.18%)	54 (9.82%)	550 (91.67%)	> 0.05
No	45 (91.49%)	5 (8.51%)	50 (8.33%)	1
Untrimmed finger				
Yes	257 (91.14%)	27 (8.86%)	284 (47.33%)	> 0.05
No	283 (89.56%)	33 (10.44%)	316 (52.67%)	1
Nail biting habit				
Yes	287 (90.54%)	30 (9.46%)	317 (52.83%)	> 0.05
No	255 (90.88%)	28 (9.12%)	283 (47.17%)	1
Have private toilet				
Yes	514 (90.37)	55 (9.63%)	569 (94.83%)	> 0.05
No	30 (96.77%)	1 (3.23%)	31 (5.17%)	1

All cases of the soil transmitted helminthes infection among school children were with light infection, such as *Hookworm*, *E. vermicularis*, *Taenia species*, *A. lumbricoide*, *T. trichiura* and *H. nana* infections (Additional file 1: Table S2).

The overall prevalence of soil transmitted helminthes infection was significantly associated with mother’s education. School children with illiterate mother were more infected than school children with University graduate mothers (p = 0.016) (Table 2). There was a significant difference in the prevalence of hookworm between boys and girls (p = 0.041) and *A. lumbricoide* (*p *= 0.037) (Additional file 1: Table S3).

### Discussion

The overall prevalence of soil transmitted helminthes was 9.5%. This result was below the results from Dembia district Northwest Ethiopia, Mizan-Aman, Chencha town and Wolaita Zone, Southern Ethiopia [17–20]. This massive improvement in the prevalence of soil transmitted helminthes might be due to the preventive chemotherapy given to the school children. Therefore, Ethiopia has the chance to achieve the 2020 strategic goals.

Reports from Chencha town Southern Ethiopia have also shown that the individual prevalence of *A. lumbricoide* and *T. trichiura* was higher than the results of this study [18] and a study from Wolaita zone Southern Ethiopia *A. lumbricoide*, *T. trichiura*, *E. vermicularis*, *Taenia* spp. and *Hookworm* was higher than the results of this study. But the result of *H. nana* was similar with this study [17]. Prevalence of helminthes species in Dembia district, Northwest Ethiopia have shown that, *A. lumbricoide* and *T. trichiura* infections were higher than this study while *hookworm* and *Taenia* species infections were lower than this study [19]. This low prevalence of *A. lumbricoide* and *T. trichiura* might be due to the control and elimination program given in the study area. Children under the age of 6 years were more infected than children above 6 years. This might be due to younger children have poor personal hygiene since they play in the soil, eat food without washing their hands and put their fingers in their mouth. Similarly, a report from Mizan-Aman town has shown that younger children were more infected with intestinal parasites [20].

With regard to associated risk factors, results from Chencha town Southern Ethiopia the presence of intestinal parasitic infections have statistically significant association with educational status of the household heads and the type of latrine, but in this study only educational status of the households was significantly associated [18]. This may be due to educated mothers may teach their children to practice personal hygiene and environmental sanitation, especially not to play in the soil, hand washing practice before meal and after defecation. A research made in Malasia have shown A school-based health education learning package (HELP) was developed which displayed a significant impact in terms of reducing soil transmitted helminthes infections [21].

Assessment of infection intensity of soil transmitted helminthes in Tanzania have shown that majority of the cases had light to moderate infection intensity, while in this study all of the cases were with light intensity [22]. This implies that one of the strategic goals of the 2020 is achieved in the study area before the expected time.

### Conclusion

The overall prevalence of soil transmitted helminthes in the study area was low (9.5%). Of the helminthes species identified, *hookworm* was the most prevalent helminthes species isolated (4.2%) followed by *A. lumbricoide* (3%). All cases of soil transmitted helminthes infections were with light intensity. This might be due to preventive mass chemotherapy given in school children. Educational status of the mother in higher education was protective from soil transmitted helminthes infections.

## Limitation of the study

This study was done among school children and the community was not included as part of the study and the laboratory diagnostic technique used in this study was Kato-Katz. It was better if two laboratory methods and more sensitive laboratory techniques such as molecular techniques and SAF-ether concentration methods were used for the diagnosis of the parasites as the prevalence of soil transmitted helminthes in the study area was low.

## Additional file

**Additional file 1.** Prevalence of soil-transmitted helminths among school children in Gurage zone Southern Ethiopia (January to December 30, 2017) (N = 600).

## Abbreviations

NTDs: neglected tropical diseases
WHO: World Health Organization
SSA: sub-Saharan Africa
STHs: soil transmitted helminthes
DALYs: disability adjusted life years
epg: egg per gram of stool
