# Supplementary material for: Prevalence, infection intensity and associated factors of soil transmitted helminths among primary school children in Gurage zone, South Central Ethiopia: a cross-sectional study design
Source: BMC Res Notes. 2019 Apr 16;12:231. doi: 10.1186/s13104-019-4254-8 (PMC6469099; doi:10.1186/s13104-019-4254-8)
Supplement: Supplementary file 1 — Additional file 1. Prevalence of soil-transmitted helminths among school children in Gurage zone Southern Ethiopia (January to December 30, 2017) (N = 600). [file 13104_2019_4254_MOESM1_ESM.docx]

Table S1: Prevalence of soil transmitted helminthes among school children in Gurage zone Southern Ethiopia (January to December 30, 2017) (N = 600)

|  | Yes  n (%) | No  n (%) |
| --- | --- | --- |
| ***Hook worm*** | 25(4.2) | 575 (95.8) |
| ***E. vermicularis*** | 5(0.8) | 595(99.2) |
| ***Taenia Spp*** | 7(1.2) | 593(98.8) |
| ***T. trichiura*** | 3(0.5) | 597(99.5) |
| ***H. nana*** | 4(0.7) | 596(99.3) |
| *A. lumbricoides* | 18(3.0) | 582(97.0) |

**Table S2.** The prevalence of the most common parasites, Hookworm and *A. lumbricoide* among school children in Gurage zone Southern Ethiopia (January to December 30, 2017) (n=600)

|  | | *Hookworm* | | | 1. *lumbricoides* | | |
| --- | --- | --- | --- | --- | --- | --- | --- |
|  |  | Yes | No | P-value | Yes | No | P-Value |
| Gender | Male | 329 | 20 | 0.041 | 7 | 242 | 0.037 |
|  | Female | 249 | 2 | 1 | 13 | 238 | 1 |

**Table S3.** Infection intensity of Soil transmitted Helminthes among school children in Gurage Zone Southern Ethiopia (January to December 30, 2017) (n=600)

| Parasites | Number of eggs of the parasites per gram of stool (epg) | | |
| --- | --- | --- | --- |
|  | Light | Moderate | Heavy intensity |
| *Hookworm* | 24 | 0 | 0 |
| 1. *Lumbricoides* | 18 | 0 | 0 |
| *E. vermicularis* | 5 | 0 | 0 |
| *Taenia species* | 7 | 0 | 0 |
| *T. trichiura* | 3 | 0 | 0 |
| *H. nana* | 3 | 0 | 0 |
